# Intratracheal Bleomycin Aerosolization: The Best Route of Administration for a Scalable and Homogeneous Pulmonary Fibrosis Rat Model?

**DOI:** 10.1155/2015/198418

**Published:** 2015-05-03

**Authors:** Alexandre Robbe, Alexandra Tassin, Justine Carpentier, Anne-Emilie Declèves, Zita Léa Mekinda Ngono, Denis Nonclercq, Alexandre Legrand

**Affiliations:** ^1^Laboratory of Respiratory Physiology, Pathophysiology and Rehabilitation, Research Institute for Health Sciences and Technology, University of Mons, 7000 Mons, Belgium; ^2^Department of Pneumology, Erasme Hospital, 1070 Brussels, Belgium; ^3^Laboratory of Histology, Research Institute for Health Sciences and Technology, University of Mons, 7000 Mons, Belgium

## Abstract

Idiopathic pulmonary fibrosis (IPF) is a chronic disease with a poor prognosis and is characterized by the accumulation of fibrotic tissue in lungs resulting from a dysfunction in the healing process. In humans, the pathological process is patchy and temporally heterogeneous and the exact mechanisms remain poorly understood. Different animal models were thus developed. Among these, intratracheal administration of bleomycin (BML) is one of the most frequently used methods to induce lung fibrosis in rodents. In the present study, we first characterized histologically the time-course of lung alteration in rats submitted to BLM instillation. Heterogeneous damages were observed among lungs, consisting in an inflammatory phase at early time-points. It was followed by a transition to a fibrotic state characterized by an increased myofibroblast number and collagen accumulation. We then compared instillation and aerosolization routes of BLM administration. The fibrotic process was studied in each pulmonary lobe using a modified Ashcroft scale. The two quantification methods were confronted and the interobserver variability evaluated. Both methods induced fibrosis development as demonstrated by a similar progression of the highest modified Ashcroft score. However, we highlighted that aerosolization allows a more homogeneous distribution of lesions among lungs, with a persistence of higher grade damages upon time.

## 1. Introduction

Idiopathic pulmonary fibrosis (IPF) is a severe form of fibrosing interstitial lung disease with unknown etiology and characterized by a progressive loss of lung function associated with dyspnea and cough. This heterogeneous pathology carries an invariable poor prognosis [1], with a median survival of less than three years from diagnosis. Over the last decade, numerous treatment options have been evaluated for IPF in large clinical trials. However, a great majority of those studies demonstrated a lack of efficacy or deleterious effects [2]. Moreover, only a minority of patients can be actually accommodated within clinical trials or with lung transplantation [3]. Therapeutic options remain thus limited, despite an increased and recent interest for new antifibrotic and anti-inflammatory agents such as pirfenidone or nintedanib, which have demonstrated efficacy in several clinical studies. Pirfenidone was further approved for medication in many countries [2].

IPF pathogenesis remains poorly understood but increased evidence suggests the involvement of complex interactions between genetic predisposition, epigenetics, environment, and comorbidities [4]. Histologically, IPF is characterized by inflammatory cell proliferation, alveolar epithelial injury, fibroblast and myofibroblast hyperplasia, and extracellular matrix deposition [1, 5, 6]. The subsequent distortion of the alveolar architecture leads to gas exchange impairment and ultimately respiratory failure and death. IPF pathological process is patchy and temporally heterogeneous, suggesting sequential injuries [6]. The inflammation process was first considered to precede fibrosis but, on the basis of later observations in animal models and the lack of efficacy of immunosuppressive therapy in patients [1, 7], the paradigm about IPF pathogenesis shifted to the idea that fibrosis could result from alveolar epithelial cell (AEC) injury and deregulated repair [1, 4]. Myofibroblasts were suggested to play a central role in this pathogenesis through extracellular matrix deposition and structural remodeling [7]. The heterogeneity of their phenotype could reflect multiple progenitors such as bone marrow or epithelial cells.* In vitro* [8] and* in vivo* [8, 9] studies supported the hypothesis that AECs could serve as a source of fibroblasts through a transdifferentiation mechanism of “epithelial-mesenchymal transition” (EMT). This phenomenon was observed during pulmonary fibrosis [7] but the principal origin of these cells is still controversial [8].

Different animal models have been developed to study the mechanisms involved in lung fibrogenesis and to evaluate potential therapies (bleomycin or fluorescein isothiocyanate administration, radiation damage, silica or asbestos instillation, transgenic mice, or viral vectors). Among these, bleomycin (BLM) administration is a widely used model and the best characterized in a variety of animals and through different routes of delivery [6]. BLM induce lung injuries via its ability to cause DNA strand breakage [10] and oxidant injury [11]. Even if the BLM-induced pulmonary fibrosis does not represent a strictly equivalent of IPF, it constitutes a polyvalent model that produces morphological alterations of lung fibrosis with a robust reproducibility [12]. It has allowed elucidating many of the biological processes involved in the pathogenesis of pulmonary fibrosis, including the contribution of TGF*β* activation [12–15]. Coupled to transgenesis, this model was useful to decipher the role of genetic factors in the development of the disease [4]. Contrary to what has been described in original studies [16], reported disadvantages of BLM intratracheal (IT) model reside in its strain-dependence in mice and its resolving nature, with a variable and self-limiting fibrosis at late time-points [6]. Repetitive intratracheal administrations of BLM were described to mimic more effectively the chronic aspects of pulmonary fibrosis [17]. However, a recent systematic study, including lung function assessment during up to 6 months after a single insult of BLM in mice, has shown persistent degree of fibrosis at late time-points, with similarities to human IPF features [18]. In addition, a recent evaluation of the activated genes after BLM administration has suggested similarities between molecular signatures obtained during the late fibrosis phase and rapidly progressing IPF [19].

IT instillation, which is the most commonly used route for BLM administration in rodent, has the advantage of its low cost and its ability to deliver a well-defined dose to the lungs. Aerosol inhalation, in contrast, could result in a deposition in the upper respiratory tract. But, the most consistent disparity between these two methods relates to the BLM intrapulmonary distribution. While aerosol inhalation allows a relatively homogeneous distribution of particles throughout the lungs, IT instillation can result in focally high doses of material or, at opposite, to nontreated lung area [20, 21]. Improvement of this point is still a matter of concern. We hypothesize that IT delivery by spraying may have the advantage of delivering a precise dose directly into the lungs and assuring a homogeneous distribution of BLM. This homogeneity could suppress the need of lesion-oriented sampling of lung tissue, simplifying and improving the sample-taking for biomolecular analyses. In the present study, we compared the time-course of histological alterations developed either by IT instillation or IT aerosolization of BLM in rats. This study reveals that aerosolization route allows a better distribution of fibrosis among lungs, with the presence of higher grade damages at later time-points.

## 2. Material and Methods

### 2.1. Animals and Treatments

All procedures met the standards of the national Belgian requirements regarding animal care and were carried out in accordance with the Animal Ethics and Welfare Committee of the University of Mons. All experiments were performed on 8-week-old male Wistar rats (about 250 g body weight) bred in our animal facility (accreditation number LA1500022). Rats were housed in cages at a room temperature (RT) of 22°C, with an ad libitum access to water and food. All efforts were made to minimize stress and animals were sedated before surgical procedure with an intraperitoneal injection of ketamine (Ketalar, Pfizer, 87.5 mg/kg of b.w.) and xylazine (Sigma-Aldrich, 12.5 mg/kg b.w.). For the present study, 47 Wistar rats received 2 IU/kg b.w. of BLM (Sanofi Aventia) intratracheally either by instillation (*n* = 22; BLM diluted in 200 *μ*L saline buffer) or by aerosolization (*n* = 25; BLM diluted in 100 *μ*L saline buffer). Sham (*n* = 17) received the vehicle only (saline buffer) and controls (*n* = 3) had no intervention. Instillation was realized by transtracheal injection using a 30 G needle at a flow of 40 *μ*L/second. Concerning intratracheal aerosolization, the oropharynx was first anesthetized using a local administration of lidocaine. A microsprayer (Model IA-1C, Penn-Century, US) connected to a High Pressure Syringue (Model FMJ-250, Penn-Century, US) was then inserted transorally into the tracheal lumen. The BLM solution was then aerosolized according manufacturer's instructions, at a rate of about 15 *μ*L/second (particle size: 16–22 *μ*m; operating pressure: 3000 psi). This procedure was realized under fiberoptic laryngoscope to visualize epiglottis and ensure a good positioning of the microsprayer. Immediately after surgery, to minimize the risk of infection, rats received an intramuscular injection of antibiotic (Sodium Cefuroxime, Zinacef, 40 mg/kg b.w., GSK). At the end of the procedure, BLM and sham animals were sacrificed by exsanguination after Sodium Pentobarbital anesthesia (intraperitoneal injection of Nembutal, 60 mg/kg b.w., CEVA, Belgium) at days 3, 7, 14, 21, or 56 after BLM/saline administration.

### 2.2. Histological Analysis

Immediately after exsanguination, a bronchoalveolar lavage (using 40 mL sterile saline buffer) was performed for further biochemical investigations. Lungs were then fixed by a transtracheal injection of a solution of Duboscq-Brasil fixator (10 mL). After ligature of the trachea and the opening of the ribcage, lungs were removed, incubated 48 hours in the Duboscq-Brasil fixator, and dehydrated. Lobes were then identified and embedded separately in paraffin. Sequential 5-*μ*m sections were made for each lobe from right and left lungs, using a Reichert Autocut 2040 microtome. Sections were then placed on silane-coated glass slides and stained with Trichrome Blue for morphological analysis. The number of total cells was calculated in the most cellularized field of 0.0625 mm^2^ (with exclusion of bronchovascular axis) using a light microscope.

#### 2.2.1. Myofibroblast Quantification

Myofibroblast staining was performed on deparaffinized and rehydrated lung sections by immunohistochemistry. Sections were immunostained using the streptavidin-biotin immunoperoxidase method (ABC method) as described in [22]. Briefly, the protocol included the following steps realized at RT in humid chamber: (1) a 1-hour incubation with a rabbit polyclonal antibody directed against *α*-SMA (smooth muscle alpha-actin; 1 : 50) (2) incubation with a biotinylated goat anti-rabbit IgG antibody (1 : 50, Abcam, UK) for 30 min, and (3) incubation with ABC complexes (Dako, Denmark) for 30 minutes. Washing steps were performed in PBS. Bound peroxidase activity was visualized by incubation with DAB (3,3-diaminobenzidine) 0.05% in PBS-0.02% H_2_O_2_. The sections were counterstained with Hemalun and Luxol fast blue and were finally mounted in a permanent medium. Controls for the specificity of immunolabeling included the omission of the primary antibody. Stained cells were numbered on ten fields of 0.0625 mm^2^ (excluding bronchovascular axis), by random sampling and using a single blind method.

#### 2.2.2. Fibrosis Quantification

*Determination of Collagen Surface*. The sections were observed on a Leitz Orthoplan microscope (10x magnification) equipped with a Ploem system for epi-illumination. Pictures were obtained by a PC-driven digital camera (Leica DC 300F, Leica Microsystems AG, Heerbrugg, Switzerland). For each of the lung regions, 3 fields of 0.3816 mm^2^ were visualized. The computer software (KS 400 imaging system, Carl Zeiss vision, Hallbergmoos, Germany) allowed the morphometric analysis of images. Percentage of surface occupied by collagen was determined by calculating the ratio between blue and nonblue pixels after exclusion of alveolar airspace.

*Modified Ashcroft Scale*. Fibrosis was quantified using a modified Ashcroft scale (grade 0 to 8) designed for a standardized fibrosis evaluation in small animals [23]. Stages 1 to 3 are characterized by the presence of alveoli partly enlarged and rarefied. Gradual fibrotic changes are observed but fibrotic masses appear from rank 4. Single fibrotic masses become confluent at stage 5. Ranks 5 and 6 are characterized by variable alveolar septa which are mostly inexistent at stage 6. Lung structure is thus severely damaged at stage 5 and mostly not preserved at stage 6. Alveoli become partly obliterated with fibrotic masses at grade 7 and complete occlusions are observed at stage 8. This procedure of fibrosis evaluation was applied on each lobe and two different counting methods were confronted. Firstly, the most affected part of the section was selected (MA-method) and secondly a random sampling was applied (RS-method). In both cases, the mean of 4 fields was calculated for each section. Each lobe section was analyzed using this procedure by two blinded observers.

### 2.3. Statistical Analysis

Results are presented as mean ± SEM. Data concerning change in body weight, total cells, collagen surface, and myofibroblasts in the BLM instillation model were submitted to an analysis of variance (ANOVA) and a post hoc Duncan's test (SigmaStat/Plot 1.0 software, Germany). Fibrosis evolution over time (modified Ashcroft score) in both BLM models was compared using an ANOVA on ranks followed by a Kruskal Wallis test. The level of fibrotic damages after BLM instillation and aerosolization in late time-points was compared by the same method. Distribution in both lungs was compared by computing absolute differences between left and right scores. Levels of significance were taken as *P* < 0.05. Interobserver agreement was evaluated with the kappa index.

## 3. Results and Discussion

### 3.1. BLM Instillation Model Allowed a Gradual Fibrosis Preceded by an Inflammatory Phase

**Figure 1:**
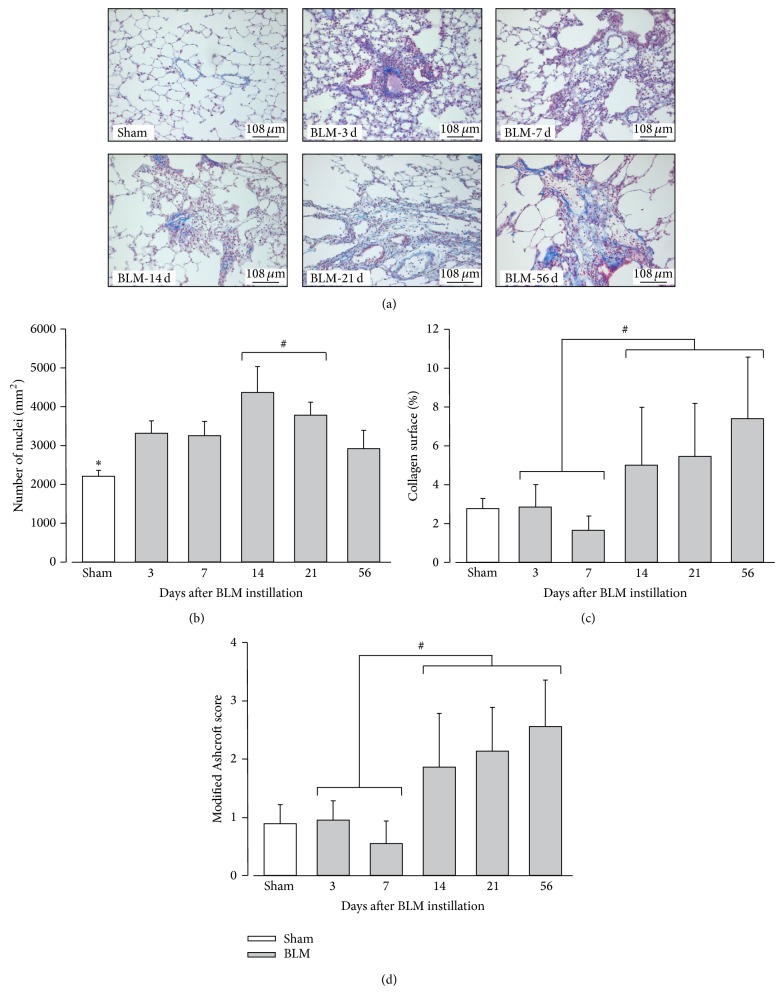
Evolution of histological alterations in rat lungs after BLM instillation. (a) Trichrome Blue staining in sham animals and in treated rats, 3, 7, 14, 21, and 56 days (3 d, 7 d, 14 d, 21 d, and 56 d) after bleomycin (BLM) instillation. Collagen is stained in blue and cells in red. Magnification: 100x. (b) Average number of cells per mm² lung surface in sham animals (in white) and 3, 7, 14, 21, and 56 days after BLM instillation (in grey). (c) Average percentage (%) of lung area occupied by collagen in sham animals (in white) and 3, 7, 14, 21, and 56 days after BLM instillation (in grey). (d) Quantification of lung fibrosis using a modified Ashcroft score in sham animals (in white) and 3, 7, 14, 21, and 56 days after BLM instillation (in grey; *n* = 3 per time-point). (b) ^∗^ *P* < 0.05 Sham versus every other time-points; ^#^ *P* < 0.05 (14 + 21 d) versus (3, 7, and 56 d); ANOVA one way followed by Duncan's test. (c-d) ^#^ *P* < 0.05 early (3–7 d) versus later (14 to 56 d) time-points; ANOVA one way followed by a Duncan's test.

**Figure 2:**
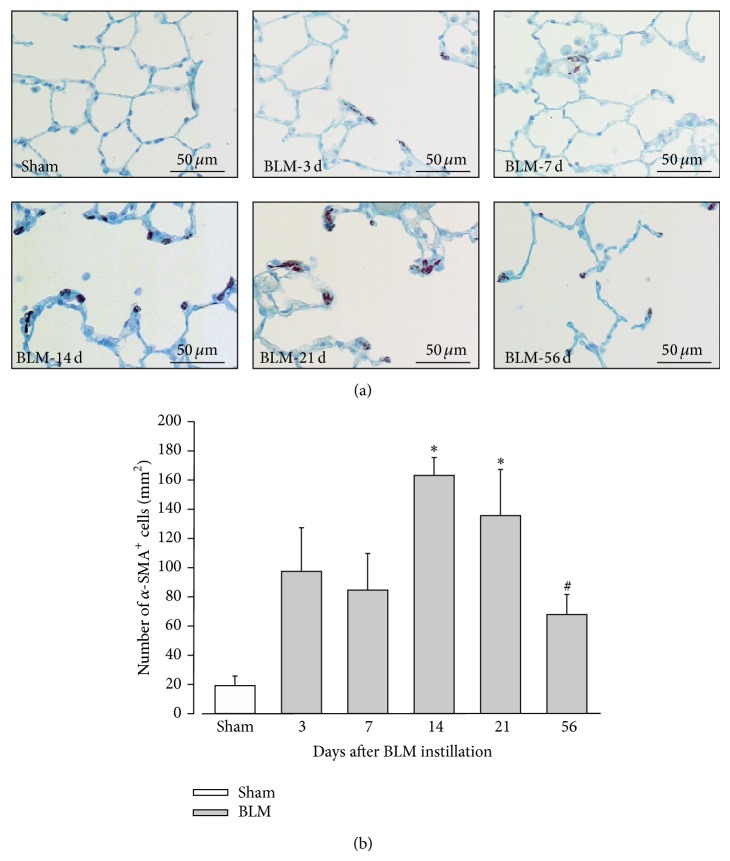
Evolution of myofibroblast number after BLM instillation. (a) Representative fields of lung sections from sham and BLM rats 3, 7, 14, 21, and 56 days after instillation. The immunohistochemistry was performed using an anti-*α*SMA (smooth muscle actin) antibody (bruin staining) and countercolored with Hemalun and Luxol blue (blue staining). (b) Average number of *α*SMA-positive (*α*-SMA^+^) cells in lung sections from sham (in white) and BLM (in grey; *n* = 3 per time-point) rats at 3, 7, 14, 21, and 56 days after instillation. ^∗^ *P* < 0.05 versus Sham; ANOVA one way followed by Duncan's Test. ^#^ *P* < 0.05 versus 14 d; ANOVA one way followed by Duncan's Test.

As reviewed in [6, 24], histological and biochemical characteristics of fibrosis are usually detectable in the BLM IT model around day 14, with a maximal response around days 21–28. However, histological damages are reported to be more variable at later time-points. Indeed, while original studies demonstrated the persistence of fibrosis for a few months, others described a resolution of the process beyond 28 days. With regards to those discrepancies, it was therefore necessary to first characterize the time-course of histological lesions after BLM instillation in our experimental conditions to facilitate subsequent comparisons. To this aim, fibrosis was assessed at 3, 7, 14, 21, and 56 days after BLM IT instillation (3–56 d) by quantification of the total cell number, percentage of surface occupied by collagen, and a modified Ashcroft score (Figure 1). Data about animal body weight, water consumption, and urine volume, measured in metabolic cages, are presented in Supplementary Material available online at http://dx.doi.org/10.1155/2015/198418 (Figure S1). Despite a slight decrease of body weight after BLM administration, no statistical difference between sham and BLM animals can be reported concerning those parameters. Macroscopic observation of lungs from BLM rats revealed the presence of atelectatic violaceous bands and white area of various sizes which alternated with apparently healthy lung tissue. Microscopic visualization confirmed the heterogeneity of histological lesions. Some lobes were totally devoid of damages and others exhibited inflammatory infiltrates centered on bronchovascular axes at day 3 and day 7. Inflammation decreased at day 14 giving rise to fibrotic lesions (Figure 1(a)). Total cell number (Figure 1(b)) was increased in BLM animals, with a maximum at days 14–21 (*P* < 0.05; 14–21 d versus 3–7 d and versus 56 d). Because these cells appeared to exhibit different morphological characteristics upon time, the presence of myofibroblasts was assessed by *α*-SMA immunostaining (Figure 2). Number of SMA-positive cells was significantly increased 14 and 21 days after BLM instillation synchronously with the beginning of fibrosis development. Then this number decreased significantly at day 56. So, the collagen-occupied surface (Figure 1(c)) and modified Ashcroft score (Figure 1(d)) were significantly different from days 14 to 56 when compared to early time-points (3–7 d). Collagen surface reached 5.0 ± 3.0, 5.4 ± 2.7, and 7.4 ± 3.2%, respectively, at 14, 21, and 56 days after BLM administration, whereas lungs from sham animals are characterized by a collagen surface of 2.7 ± 0.5%. A modified Ashcroft score ranging between ranks 2 and 3 at later time-points indicated the presence of fibrotic changes accompanied by partly enlarged and rarefied alveoli [23].

In accordance with the literature, BLM IT instillation leads firstly to an inflammatory phase that precedes a gradual development of fibrosis, with a transition occurring around day 14 after BLM delivery. Although the inflammatory process was not investigated specifically in our study by cell counting, total protein measurement in bronchoalveolar fluid, or lung TGF*β* expression, inflammatory infiltrates were observed at early time-points. Total cell number mostly reflects alveolar inflammatory cells or active fibrosis, before and after day 14, respectively. This time-point is characterized by a significantly higher number of total cells, including in particular myofibroblasts, consistent with their previously reported role in collagen deposition [7, 25]. In our experimental conditions, fibrosis characteristics can be observed until 56 days after BLM instillation. The resolution of the process is therefore not observed at this time-point, even if its activity was decreasing based on the myofibroblast number. Further studies in later time-points are necessary to elucidate discrepancies about the resolving nature of this model. Via this route of administration, fibrotic lesions were, however, heterogeneously distributed, hampering interpretation of subsequent molecular analysis or evaluation of therapeutic strategies based on a random tissue-sampling.

### 3.2. BLM IT Aerosolization Leads to a Progressive and More Homogeneously Distributed Fibrosis

**Figure 3:**
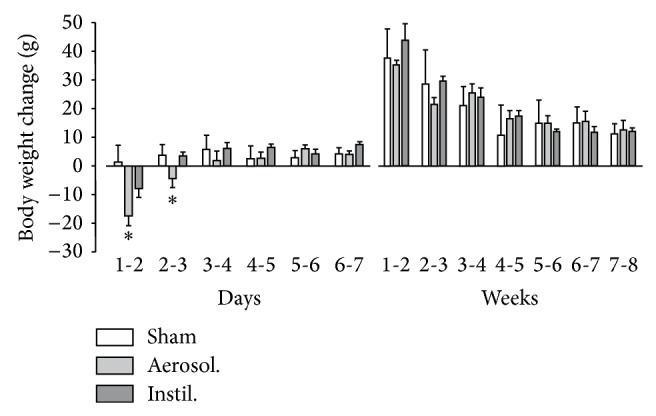
Time-course of body weight change after BLM administration. Data are represented as mean ± SEM for the sham group (*n* = 17) and after BLM aerosolization (*n* = 25) or instillation (*n* = 22). Observations were realized the first week daily and weekly then after up to day 56. ^∗^ *P* < 0.05 Aerosol. versus Instil. and Sham; ANOVA one way followed by Duncan's Test.

**Figure 4:**
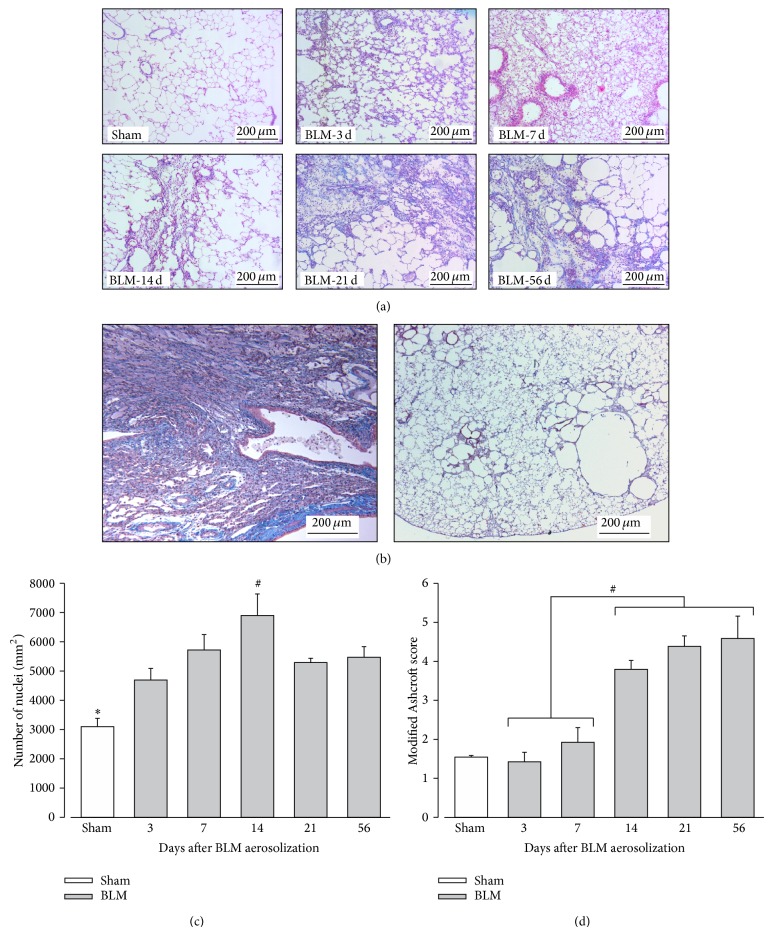
Evolution of pulmonary histopathological alterations after BLM aerosolization. (a) Trichrome Blue staining in sham animals and 3, 7, 14, 21, and 56 days (3 d, 7 d, 14 d, 21 d, and 56 d) after bleomycin (BLM) aerosolization. Collagen is stained in blue and cells in red. Magnification: 100x. (b) Left panel: peribronchial lesions are present at late time-points (days 14, 21, and 56) as after BLM instillation. Right panel: destructive lesions are observed at days 14 and 56. (c) Average number of cells per mm² lung surface in sham animals (in white) and 3, 7, 14, 21, and 56 days after BLM aerosolization (in grey). (d) Quantification of lung fibrosis using a modified Ashcroft score in sham animals (in white) and 3, 7, 14, 21, and 56 days after BLM aerosolization (in grey; *n* = 5 per time-point). (c) ^∗^ *P* < 0.05 Sham versus every other time-points; ^#^ *P* < 0.05: 14 d versus 3 d and 21 d; ANOVA one way followed by Duncan's test. (d) ^#^ *P* < 0.05 early (3–7 d) versus late (14 to 56 d) time-points; ANOVA one way followed by a Duncan's test.

**Figure 5:**
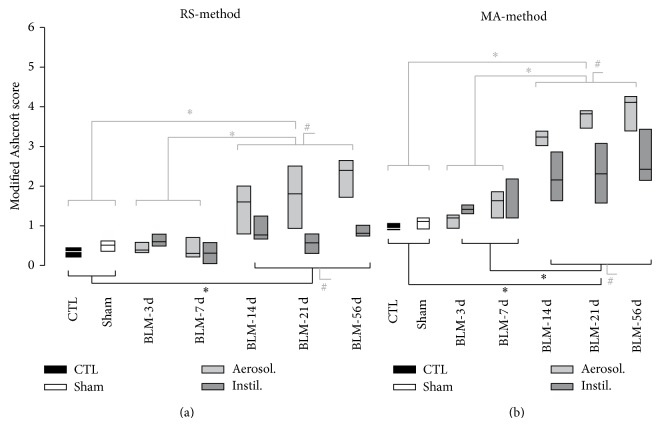
Comparison of fibrosis evolution after BLM instillation or aerosolization. Fibrosis was quantified in each lung lobe using a modified Ashcroft score, in control (CTL, in black), sham (in white), and BLM animals, at 3, 7, 14, 21, and 56 days after BLM instillation (Instil., in dark grey) or aerosolization (Aerosol., in grey). Two different methods were applied for quantification, as described in Section 2: RS- (random sampling-) method (a) and MA- (most affected field-) method (b). For statistical analysis, data from CTL and sham animals were grouped (CTL + sham) as well as results concerning BLM rats at early (3–7 d) and late (14 to 56 d) time-points. Grouped means are not different as compared by a Mann Whitney rank sum tests (CTL versus Sham) or using Kruskal Wallis one way analysis (3 versus 7 d; 14 versus 21 versus 56 d). Groups were compared as indicated, using a Kruskal Wallis one way (pairwise multiple comparison of means, Dunn's method). CTL: *n* = 3; Sham: *n* = 17; BLM Instil.: *n* = 22; BLM Aerosol.: *n* = 25. ^∗^ *P* < 0.001: late versus early versus CTL + sham; ^#^ *P* < 0.001: Aerosol. versus Instil. versus CTL + sham at late time-points. Kappa index representing interobserver agreement was for the instillation model of 0.12 and 0.09 (RS-method and the MA-method, resp.) and for the aerosolization model 0.64 and 0.43.

To improve distribution of fibrotic damages in the BLM model, we compare IT instillation to IT aerosolization of this drug. As a preliminary test, macroscopic analysis after Lissamine Green IT aerosolization has shown a homogeneous distribution of the dye among lungs. Histological alterations were then assessed after 3, 7, 14, 21, and 56 days after either BLM IT instillation or aerosolization. Data about animal body weight, water consumption, and urine volume in aerosolized animals are presented in Supplementary Material (Figure S2). As presented in Figure 3, we note that weight loss upon the two first days after BLM delivery was more pronounced in aerosolized rats as compared to instilled animals (*P* < 0.005). Microphotographs of the most representative pulmonary lesions at each time-point are illustrated in Figure 4 as well as the total cell number and modified Ashcroft score in the aerosolized group. Comparison of fibrosis quantification using the modified Ashcroft score in both models is presented in Figure 5. As described after BLM instillation, inflammatory infiltrates were observed at early time-points after BLM aerosolization (Figure 4(a), 3–7 d), followed by a transition at day 14 to a fibrosis state. Total cell number reached a peak at this particular time-point (Figure 4(c)). In addition, perilesional emphysema (Figure 4, 14–56 d) and peribronchic lesions (Figure 4(b)) were present in both models at late time-points (14 to 56 d). A gradual increase of fibrotic changes was observed, reaching a significantly higher modified Ashcroft score at late time-points in both models, as compared to sham animals (Figure 5). Modified Ashcroft score differed between late and early time-points in both models when fields from the most affected part of each lobe were considered for quantification (MA-method, Figure 5(b)). Values obtained were on average 1.3 ± 0.1 in shams and 1.8 ± 0.1, 1.9 ± 0.4, 2.7 ± 0.3, 2.8 ± 0.5, and 3.2 ± 0.4 in the instillation groups at days 3, 7, 14, 21, and 56, respectively. Corresponding values after aerosolization were 1.4 ± 0.1, 1.9 ± 0.2, 3.8 ± 0.1, 4.4 ± 0.12, and 4.6 ± 0.3 at the same time-points in BLM animals.

When randomly chosen fields were considered (RS-method, Figure 5(a)), the modified Ashcroft scores from late time-points were significantly higher compared to early time-points in aerosolized BLM animals but not in IT instilled rats. So, the mean value of the modified Ashcroft score for the aerosolized group reached 2.3 ± 0.2 in the later time-point (day 56) but only 0.9 ± 0.1 for the instilled group. These results could be explained by the presence of more focal lesions in lungs from instilled animal. Indeed, in average, lesions appeared more moderate when fields were randomly chosen, likely due to the presence of lung area without any fibrotic lesion.

Data from both quantification methods therefore indicate a more homogeneous distribution of fibrotic lesions after IT aerosolization as compared to IT instillation. This was previously described in a rabbit model of fibrosis consisting in BLM intranasal nebulization [26]. This route was also shown to allow a more homogeneous distribution of material into the lungs in different species including mice [20, 21]. The oropharyngeal aspiration is another method often used in mice and consisting of pipetting BLM into the back of the oral cavity [27, 28]. Gravity and natural inhalation by the animal draw the liquid into the lung [29]. This method was shown to lead to a better distributed fibrotic area among lungs in mice and rats, compared to the intranasal method [30]. IT aerosolization using a sprayer has, however, the advantages of (i) providing a more direct access into the lungs and avoiding material loss in the upper respiratory tract and (ii) delivering solutions as microdroplets allowing a more peripheral and diffused material deposition as compared to liquids. In rats, procedures for intubation and aerosol delivery were described in [31, 32]. The usefulness of this noninvasive endotracheal route was demonstrated in mice by delivery of a suspension of fluorescent nanospheres [33]. IT aerosol delivery was therefore used to administer BLM in mice to model lung fibrosis [19, 34] and, in another context, to deliver siRNAs to modulate lung immunopathology in a murine model of tuberculosis [35, 36]. However, in mouse and especially in rats, IT instillation, rather than spraying, remains a frequently used route for BLM models [37–39].

**Figure 6:**
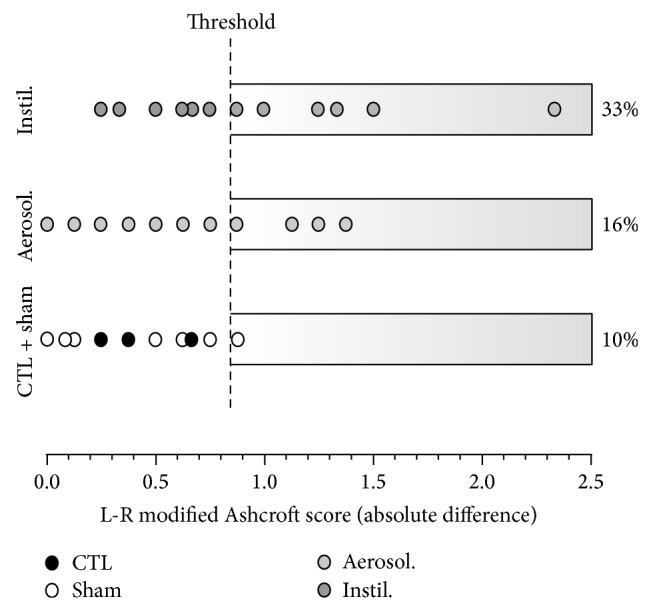
Difference between left and right lung fibrotic lesions after BLM aerosolization or instillation. This graph represents the modified Ashcroft score absolute difference between L and R lungs, for control and sham animals (CTL + sham; in white) and after BLM aerosolization (Aerosol., in grey) or instillation (Instil., in dark grey) at all time-points. Quantification was made using the MA-method by two observers. Each circle represents the mean between values obtained by both observers for each animal. The threshold was fixed to include 90% of the values of the CTL + sham group (confidence interval calculated as mean ± 1.66 SEM). 16% and 33% of the values are above this threshold for instilled and aerosolized animals, respectively. CTL + sham: *n* = 20; BLM Instil.: *n* = 22; BLM Aerosol.: *n* = 25.

In addition, our study reveals that interobserver agreement was better after aerosolization than instillation. So, in the aerosolized group, the agreement was moderate or substantial depending on the method used (MA or RS), whereas the kappa index disclosed only to a slight agreement for the instillation group whatever the field selection. The difference of fibrotic-lesion distribution between lungs (absolute difference between modified Ashcroft score in left and right lungs) is represented at Figure 6. Data outside the 90% confidence interval calculated from controls and sham values are 3 times more frequent in the instilled than in the aerosolized groups. Aerosolized animals exhibited a moderate difference between right and left lungs in terms of fibrosis, with only 16% of the values above the threshold (instead of 10% for controls and sham animals).

### 3.3. BLM IT Aerosolization Allowed the Persistence of More Severe Fibrotic Lesions upon Time

The increased loss of body weight induced by the aerosol method (Figure 3) suggests that pulmonary lesions have a higher systemic effect at least during the first few days after treatment. Moreover, as presented in Figure 5, the modified Ashcroft scores at late time-points (14–56 d) were significantly higher in the aerosolized groups independently of the quantification method used. When the RS-method is considered (Figure 5(a)), this difference could be explained by the presence of unaffected area in instilled lungs leading to a lowering of the mean Ashcroft score. However, on average, a higher score was also observed in the most affected lung area (MA-method) from aerosolized animals (Figure 5(b)) indicating the development of a more severe fibrosis (modified Ashcroft score between 4 and 5) upon using this route of administration. In accordance with these data, we note that destructive lesions are only observed in the aerosolization group at days 14 and 56 (Figure 4(b)). We suggest that, with aerosolization, a better penetration of BLM into small airways and more scattered AECs alterations could lead to an amplified myofibroblastic stimulation and a subsequent increase in fibrotic tissue deposition. At the opposite, with instillation, the overwhelming of the alveoli could also decrease the oxygen pressure in the vicinity of BLM molecules and thereby reduce its toxicity. Further studies will be necessary to clarify the relationship between increased fibrotic damages at later time-points and more dispersed initial alterations.

## 4. Conclusion

Both intratracheal instillation and aerosolization of BLM induce the development of fibrosis following an initial inflammatory phase. However, the fibrotic process is more localized after BLM instillation and is restricted to overwhelmed area, which is a major drawback for the tissue sampling. In the present study, we demonstrate that the IT aerosolization route allows a more homogeneous distribution of fibrosis and is associated with more severe lesions upon time. As compared to intranasal delivery, the IT spraying allows the delivery of a precise dose, avoiding drug loss in upper respiratory tracts.

Finally, it is necessary to consider whether BLM rodent models could be directly applicable to human IPF. In terms of histological alterations, both conditions result in the development of fibroblastic foci. In IPF, their location is heterogeneous and mostly basal and subpleural. In contrast, lesions are initially bronchocentric in several BLM IT models, although our conditions led to more peripheral damages. Moreover, BLM rodent models and IPF do not share a similar pattern of development and progression, and functional assessment has to be further realized to better understand similarities and differences between the two pathological states (reviewed in [24]).

In conclusion, despite the fact that BLM delivery in rodent does not perfectly reproduce IPF, it still constitutes a well-characterized model of pulmonary fibrosis which is still widely used today. IT aerosolization is a good alternative to the instillation method, allowing a homogeneous fibrosis thus limiting sample-dependent variability for subsequent biochemical analysis or testing of new therapeutic options.

## Supplementary Material

Supporting Material represents time course of body weight, daily consumption of water and urinary volumes, measured using metabolic cages for sham and BLM animals. Figure S1 is related to the BLM instillation group and figure S2 concerns the BLM aerosolization group.
