# Supplementary figures and images for: Intratracheal Bleomycin Aerosolization: The Best Route of Administration for a Scalable and Homogeneous Pulmonary Fibrosis Rat Model?
Source: Biomed Res Int. 2015 May 3;2015:198418. doi: 10.1155/2015/198418 (PMC4433632; doi:10.1155/2015/198418)

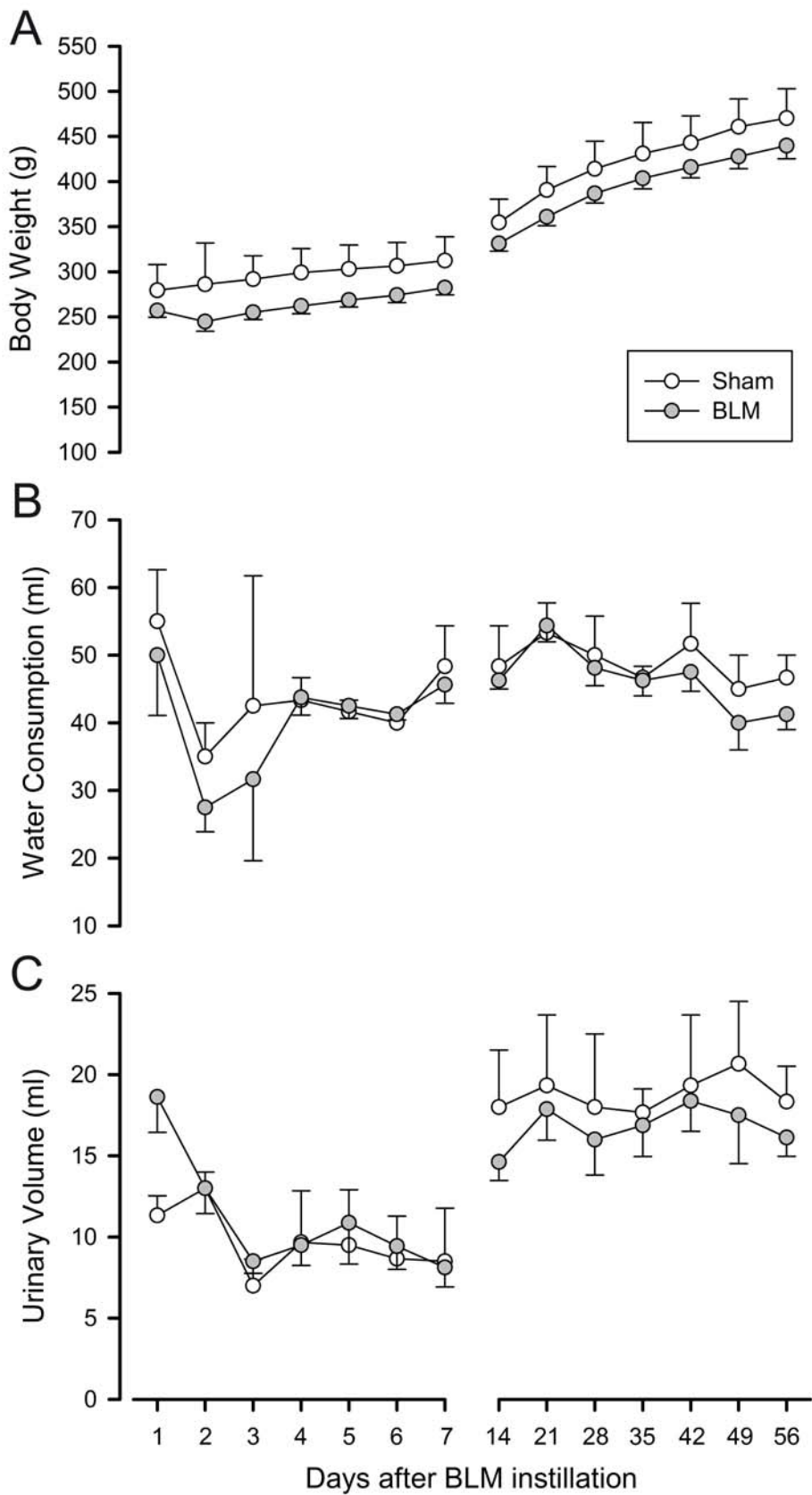

Supplement: Supplementary file 1 — Supporting Material represents time course of body weight, daily consumption of water and urinary volumes, measured using metabolic cages for sham and BLM animals. Figure S1 is related to the BLM instillation group and figure S2 concerns the BLM aerosolization group. [file 198418.f1.pdf]

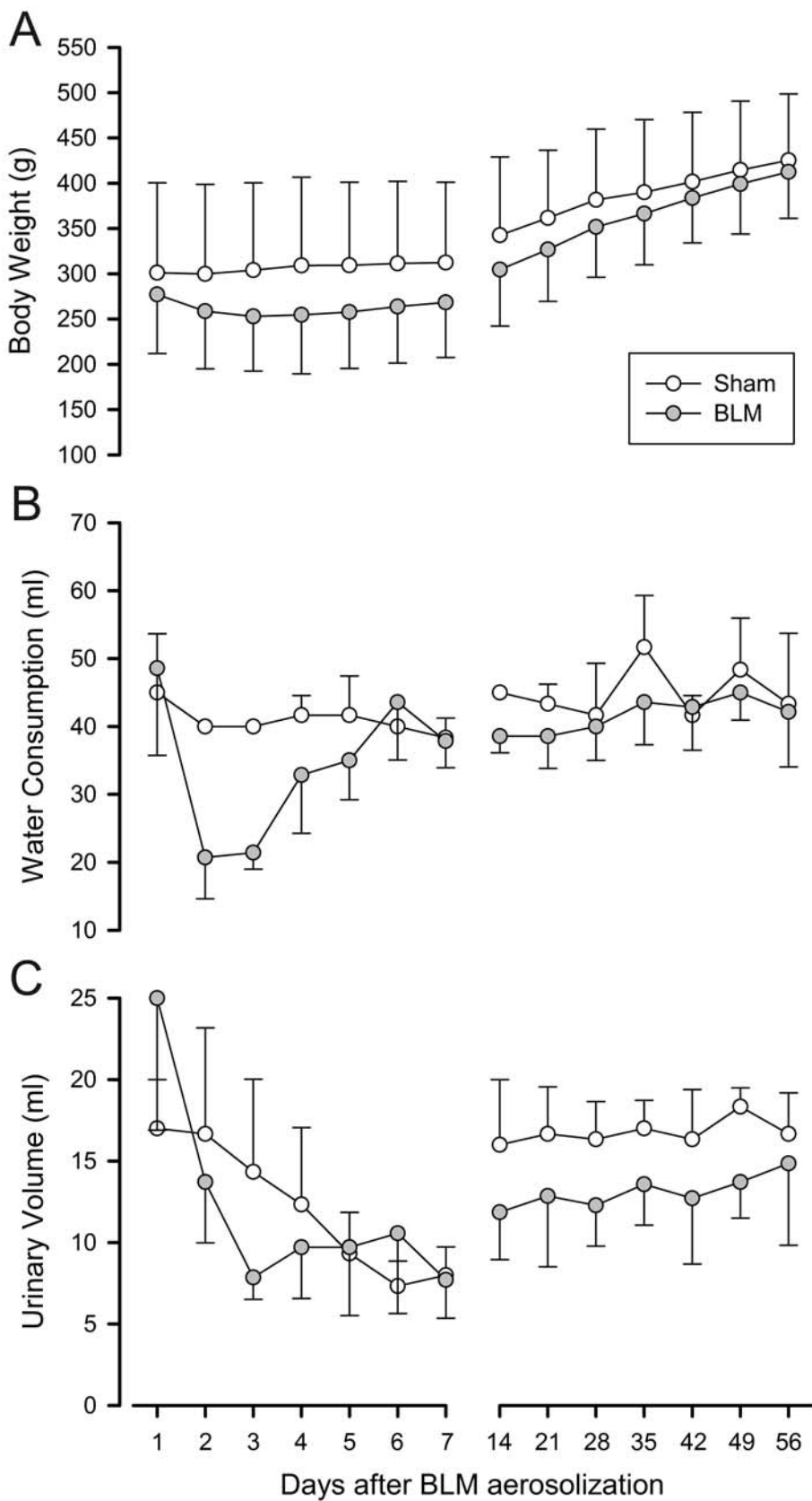

Supplement: Supplementary file 2 [file 198418.f2.pdf]
